# Optical Dielectric Loss as a Novel Approach to Specify the Types of Electron Transition: XRD and UV-vis as a Non-Destructive Techniques for Structural and Optical Characterization of PEO Based Nanocomposites

**DOI:** 10.3390/ma13132979

**Published:** 2020-07-03

**Authors:** Dana S. Muhammed, Mohamad A. Brza, Muaffaq M. Nofal, Shujahadeen B. Aziz, Sarkawt A. Hussen, Rebar T. Abdulwahid

**Affiliations:** 1 Department of Physics, College of Basic Education, University of Sulaimani, Qlyasan Street, Sulaimani 46001, Iraq; dana.muhammad@univsul.edu.iq (D.S.M.); rebar.abdulwahid@univsul.edu.iq (R.T.A.); 2 Department of Manufacturing and Materials Engineering, Faculty of Engineering, International Islamic University of Malaysia, Kuala Lumpur, Gombak 53100, Malaysia; mohamad.brza@gmail.com; 3 Hameed Majid Advanced Polymeric Materials Research Lab., Department of Physics, College of Science, University of Sulaimani, Qlyasan Street, Sulaimani 46001, Iraq; sarkawt.hussen@univsul.edu.iq; 4 Department of Mathematics and General Sciences, Prince Sultan University, P.O. Box 66833, Riyadh 11586, Saudi Arabia; muaffaqnofal@gmail.com; 5 Department of Civil Engineering, College of Engineering, Komar University of Science and Technology, Sulaimani 46001, Iraq

**Keywords:** PEO polymer nano-composite, SnTiO_3_ nano-particles, XRD, UV-vis analysis, optical properties

## Abstract

The structure and optical properties of polyethylene oxide (PEO) doped with tin titanate (SnTiO_3_) nano-filler were studied by X-ray diffraction (XRD) and UV-Vis spectroscopy as non-destructive techniques. PEO-based composed polymer electrolytes inserted with SnTiO_3_ nano-particles (NPs) were synthesized through the solution cast technique. The change from crystalline phase to amorphous phase of the host polymer was established by the lowering of the intensity and broadening of the crystalline peaks. The optical constants of PEO/SnTiO_3_ nano-composite (NC), such as, refractive index (*n*), optical absorption coefficient (*α*), dielectric loss (*ε*_i_), as well as dielectric constant (ε_r_) were determined for pure PEO and PEO/SnTiO_3_ NC. From these findings, the value of *n* of PEO altered from 2.13 to 2.47 upon the addition of 4 wt.% SnTiO_3_NPs. The value of ε_r_ also increased from 4.5 to 6.3, with addition of 4 wt.% SnTiO_3_. The fundamental optical absorption edge of the PEO shifted toward lower photon energy upon the addition of the SnTiO_3_ NPs, confirming a decrement in the optical band gap energy of PEO. The band gap shifted from 4.78 eV to 4.612 eV for PEO-doped with 4 wt.% SnTiO_3_. The nature of electronic transitions in the pure and the composite material were studied on the basis of Tauc’s model, while optical *ε*_i_ examination was also carried out to calculate the optical band gap.

## 1. Introduction

Over the last three decades, polymer materials have been studied extensively for their potential applications, with a focus on developing various polymers to replace the utilization of metals [1]. For this purpose, several strategies have been established for the synthesis of many advanced polymer nano-composites (NCs) using semiconductors and metals [2]. During these decades, several common types of polymers have been explored, for instance, poly (vinyl chloride) (PVC), poly (methyl methacrylate) (PMMA), poly (ethylene oxide) (PEO), and poly (vinylidene fluoride) (PVDF) [3]. Modification of the optical, electronic, and structural properties of inorganic-organic nano-particles (NPs) can be carried out via manipulation of the particle dimensions without changing the chemical composition. Enhancement of properties of the host polymer via NP synthesis relies on the shape, size, composition, and structure of the NPs [4,5]. These enhancements have particular impact on the optical properties, namely, light absorption, reflection, antireflection, and polarization [6,7,8,9]. The superiorities of polymer materials derive from their transparency, cost-effectiveness, ease of processing, light weight, and satisfactory mechanical properties. It is also well established that the polymer of PEO is one of the most appropriate candidates for application in the optics field. However, these materials normally exhibit relatively low refractive indices [10,11].

A polymer that encompasses more than one desired property as a result of various modifications can perform multiple functions. These modifications, especially of the polymer’s structure and optical properties involve addition of nano-size materials. Addition of dopants reduces the energy gap as a consequence of changing the mechanism of electron transitions [12]. PEO-based materials are promising polymer candidates because of their relatively high thermal stability. PEO is a semi-crystalline material that contains a crystalline phase as well as amorphous phase at ambient temperature [12,13]. Several studies have confirmed that PEO has unique properties. The outstanding properties are sufficient dimensional stability, high capacity to encompass salts, relatively high ionic conductivity in the amorphous region, resistance to corrosion, relatively low cost, convincing mechanical flexibility, and chemical stability [14,15,16]. In material design, to gain insight into the band gap structure and band gap energy in non-crystalline as well as crystalline materials, it is critical to take into consideration the optical absorption spectra [17,18]. On the one hand, absorption spectra examination in the low energy region provides details regarding atomic assignment of the fundamental vibrations; on the other hand, analysis of the spectra in the high energy region offers insight into the electronic levels in atoms [19]. Modification of the optical properties is achieved by insertion of dopants into polymer matrices [20]. As a principle, when UV and visible photons interact with materials, three phenomena occur: absorption, transmission, and reflection. Based on theoretical and practical considerations, a direct relation between these phenomena has been exhibited where the material’s absorption coefficient (*α*) and refractive index (*n*) are decisive [18].

Much research effort has been devoted to inspect the optical characteristics of doped host polymer matrices [21], for instance, PVA polymer incorporated with Cu(II)-complex [22], PEO polymer treated with TiO_2_nano-filler [23], and doped with ZnO NPs [24]. In a previous work, the optical properties of solid polymer doped with PbTiO_3_ and Pb(ZrTi)O_3_ NPs have been examined. However, due to the health-hazardous nature of Pb and its detrimental impact on the environment, there is a strong motivation to replace Pb with environmentally friendly elements [25]. One of the promising Pb-free materials that has recently been studied is SnTiO_3_ that is theoretically indicated to possess a large dielectric constant (ε_r_) as computed via the study of first-principle [25,26,27]. Therefore, Sn-based materials were recognized as a promising replacement for Pb-based materials in future use in devices such as piezoelectric transducers, power harvesters, non-volatile memories, and optical waveguides [28,29,30]. The replacement of the PbTiO_3_ A-site with Sn allows us to obtain analogous material which is more eco-friendly.

In this report the optical characteristics of PEO doped with SnTiO_3_ NPs are investigated. Environmental concern regarding hazardous elements has encouraged researchers to discover more efficient and eco-friendly materials. This material should have big electric polarization which is reliant on the structure of the perovskite and relatively high *n* [11,31]. Furthermore, SnTiO_3_ material possesses elastic properties which are vital for basic familiarization with inter-atomic potentials (inter-atomic bonding). As a consequence, this material exhibits relatively high ε_r_ [32,33]. The goal of this study is to examine the optical and structural characteristics of a NC solid polymer. In the current study, enhancements of optical characteristics of the PEO/SnTiO_3_nano-composite have been achieved, namely, relatively high *n,* ε_r_, and transparency. It is also shown that the current methodology might offer an alternative for the band gap energy and precisely determining the kind of electronic transitions.

## 2. Experimental Detail

### 2.1. Polymer Composite Preparation

Polyethylene oxide (PEO) was employed as the host polymer in this study. The PEO (molecular weight> 5 × 10^6^ g/mol) powder material was provided by Sigma-Aldrich. Solution-casting method was used in the films’ preparation. The PEO solution was prepared by adding one gram of PEO powder to distilled water (50 mL) and stirring through a magnetic stirrer for 5 h at room temperature. Once the polymer solution was obtained in the form of a clear viscous solution, a portion of 4 wt.% of SnTiO_3_, supplied by Sigma-Aldrich, was added to it. Then, the solution was stirred continuously to prepare the PEO/SnTiO_3_ polymer NC. The pure PEO and PEO/4 wt.% of SnTiO_3_ were labeled as PESNT0 and PESNNT1, respectively. Ultimately, the homogenous solutions were cast into plastic Petri dishes and permitted to dry at room temperature. Later, the films were placed in a desiccator containing blue silica gel for additional drying prior to characterizations.

### 2.2. Characterization Techniques

#### 2.2.1. X-ray Diffraction

The structural characterizations and the impact of the nano-size filler for all the samples were assessed by means of X-ray diffraction (XRD) and UV-Vis spectroscopy. An X-ray diffractometer (PANalytical, Almelo, The Netherlands) with a working current of 40 mA and a working voltage of 40 kV was used for acquiring the patterns of XRD. The samples were examined with a monochromatic CuKα X-ray radiation beam with a wavelength (λ) of 1.5406 Å plus a glancing angle (2θ) in the range of10° to 80° with 0.1° step size at room temperature.

#### 2.2.2. UV-Vis Measurement

UV-VIS spectroscopy is employed to measure the light absorption of liquid and solid samples through the wavelength range of ultraviolet and optical. It is an influential technique to find the optical properties of samples such as transmittance, absorbance, and reflectance. An ultraviolet-visible (UV-Vis) spectrometer (V-570, Jasco, Japan) with the wavelength in the range between 190 and 790 nm was employed to obtain the UV-Vis absorption spectra of the fabricated samples. For this purpose, the prepared samples were cut with dimensions of (1 × 2 cm^2^) in order to fit into the sample holder. Then, the spectrometer was calibrated to subtract the air absorption. The samples’ thicknesses were measured and found to be in the range of119 to 122 µm.

## 3. Results and Discussion

### 3.1. X-Ray Diffraction (XRD) Analysis

**Figure 1:**
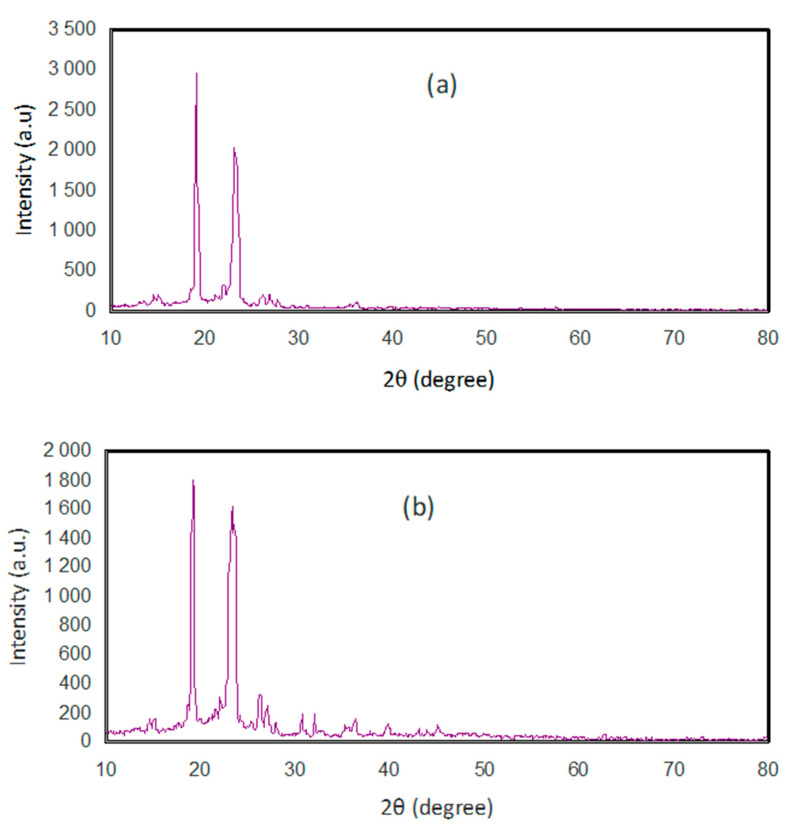
XRD pattern of for (**a**) PESNT0 and (**b**) PESNT1 composite films.

Figure 1 indicates the XRD spectra for the pure PEO and the composite film. Figure 1a exhibits the pure PEO XRD pattern. The appearance of two narrow peaks illustrates the dominance of the crystalline phase [34]. The two characteristic peaks centered at 18° and 24° and weak peaks (i.e., low intensities) at higher-angle degrees. It is fascinating to perceive that the intensity of the peaks decreases and they become broad in the presence of the nano-size SnTiO_3_ as shown in Figure 1b. This implies that the amorphous region in PEO increases at the expense of the crystalline phase. This could be a result of the interaction that occurred between the SnTiO_3_ and PEO. It also reveals the dominance of the amorphous phase facilitated by polymer chain segmental motion [35].

These results emphasize that the decrease in the crystalline phase is due to the lowering of the compact nature of the polymer body [36,37]. It was found that the PEO building units such as C–H bond, C–C bond, and C–O bond provide a good crystalline and electrochemical stability nature [36,38]. The PEO is found to exhibit as a linear and semi-crystalline polymer with distinguishable diffraction peaks [38,39]. It is well known that the structural properties of the pure PEO can be altered through both blending and adding additives. The literature documented that the decrease in crystalline nature of PEO after blending and adding NPs are ascribed to a strong interaction between the host polymer and the additives [40,41,42,43,44]. This supports the results obtained in this study and confirms the interaction occurred between the PEO polymer and the nano-size SnTiO_3_.

### 3.2. Optical Properties

**Figure 2:**
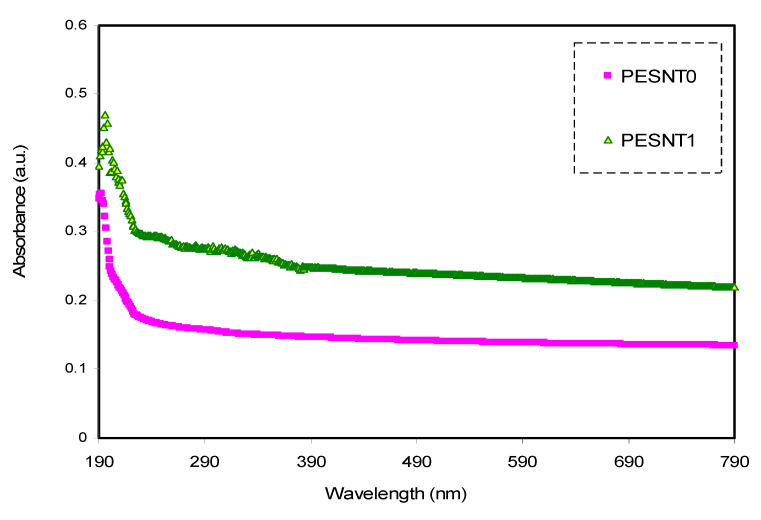
Pure PEO (PESNT0) and PESNT1 composite samples’ optical absorption spectra.

Examinations of optical absorption for pure PEO as well as PEO doped with SnTiO_3_ films were conducted to find out more concerning the band structure alterations and the optical energy band gap determination at room temperature. Analysis of optical absorption spectra was employed to determine the optically induced transitions and gain details regarding the films’ band structures [45,46]. The absorption spectra of UV-Vis for pure PEO as well as PEO/SnTiO_3_ films are shown in Figure 2. It can be seen that the pure PEO film has no noticeable absorption peak in the 200 and 380 nm wavelength. However, in the UV region there is a broad absorption band for the addition of 4 wt.% SnTiO_3_ into the PEO sample. It is also perceived that there is a small change of the peak in the PEO/SnTiO_3_NC sample to a higher wavelength with peak broadening (i.e., low intensity) compared to pure PEO.

It is well-known that when the energy of the emitted photons is lower than the energy difference between the two levels, the photons’ energy is not absorbed and the material is transparent to the photons. Conversely, in the case of absorption of photons with enough high energy, the valence electron transport between the two energy states are observed [47]. The absorption spectrum plateau region in the optical ranges indicates that the PEO composite is transparent to the visible photons while electrons are incapable of jumping within the band structure [48].

**Figure 3:**
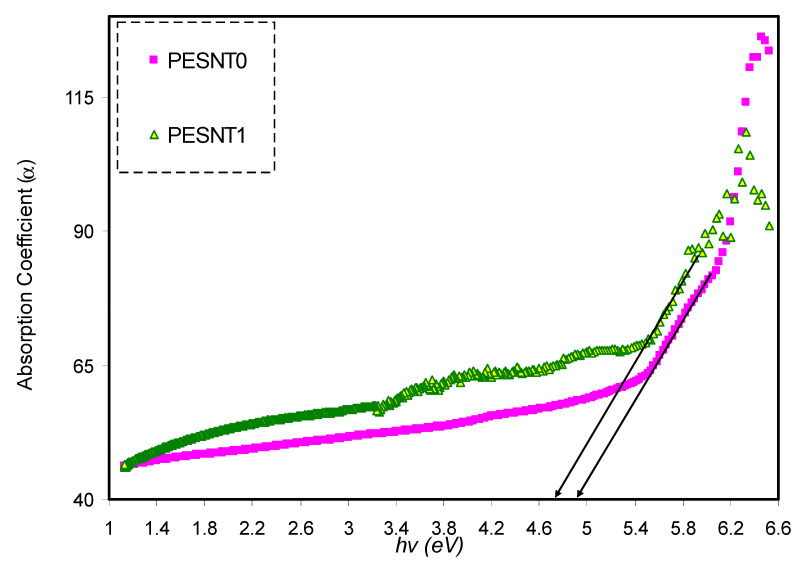
Optical absorption coefficient (*α)* against photon energy of pure PEO (PESNT0) and PESNT1 composite samples.

The optical absorption coefficient (*α*) as opposed to wavelength (λ) is described as the power portion, which is absorbed per unit length of a medium. The *α* is assessed from the absorption data investigation using the giving equation [6,36,49]

(1) $$\alpha (\lambda )=2.303\left(\frac{A}{d}\right)$$

where *d* and *A* are the thickness of the samples and the absorbance, respectively; then *α* can also be defined as a measure of the ability of materials to absorb light photons [50]. Optical absorption examination offers insight into the solid’s band structure. The *α* against photon energy for pure PEO as well as PEO inserted with SnTiO_3_ is shown in Figure 3. It is noticeably evident that addition of nano-size SnTiO_3_ into the PEO causes an absorption edge shift to lower photon energy. In general, semiconductors and insulators are categorized into two main groups: direct band gap (DBG) group and indirect band gap (IBG) group. For DBG semiconductors, the top of the valance band (VB) and bottom of the conduction band (CB) coincide at similar values of *k*. In contrast, in IBG semiconductors, the top of VB and bottom of CB do not coincide at similar values of *k.* Transition from VB to CB in materials of IBG groups has to be in relation with a phonon (lattice vibration energies) of the crystal momentum [20]. To determine the absorption edge position, the *α* linear part as opposed to the curve of *hυ* can be extrapolated to zero value of absorption [6]. The trap levels development inside the optical band gap results in shifting of the optical absorption edge. As a consequence, electrons cross the VB at the top to the CB at the bottom through these new levels [51,52]. The current optical measurement results are comparable to those obtained for PEO by Kumar et al. [3].

### 3.3. Refractive Index and Optical Dielectric Constant Study

**Figure 4:**
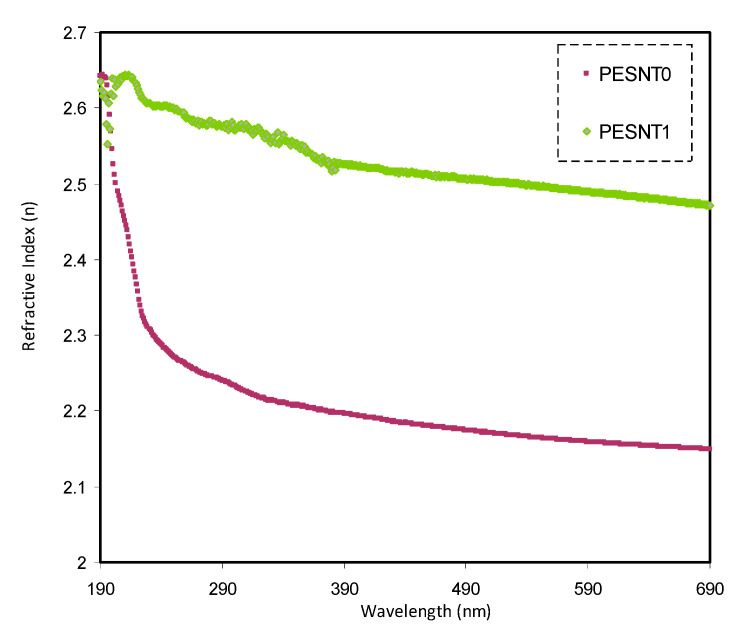
Refractive index (*n*) of pure PEO (PESNT0) and PEO:SnTiO_3_ (PESNT1) samples.

Another decisive parameter of optical phenomena is the refractive index (*n*) that is governed by the reflection coefficient (*R*) and extinction coefficient (*k*). The *n* value is dependent on the wavelength of incident photon as illustrated in Figure 4. The current study, has confirmed that the *n* of PEO increases with addition of SnTiO_3_ NPs. Aziz et al. [18] indicated that the*n* of the polymer films is characterized by a dispersion region at the low wavelength of the incident photons and a plateau region at the high wavelength of the incident photons [53]. Figure 4 indicates that the *n* of the PEO is modified by addition of SnTiO_3_, with the *n* value changing from 2.18 to 2.50. These values have been obtained at the plateau region. *n* is the decisive optical communication factor when using a material in a specific optoelectronic application [18,54]. It is important that the optical characteristics are dependent on the electronic band structure and atomic structure of materials [18]. Herein, the electronic structure of the PEO matrix is drastically modified via addition of SnTiO_3_ NPs. The *n* of materials is formulated on the basis of*R* and *k* by the Fresnel formulae [55,56,57]:

(2) $$n=\left(\frac{1+R}{1-R}\right)+\sqrt{\frac{4R}{{\left(1-R\right)}^{2}}}-k^{2}$$

The *R* denotes the reflectance. The *k* stands for the extinction coefficient. Thus, when the *k* is smaller than the *n* this means, to a large extent, transparency of the composite samples [58]. It is worth mentioning that the *n* depends upon both the polarizability and density of the composite. Density here refers to the extent of the electron population; when the density is high, the interaction of light with the exposed material increases. Meanwhile, polarizability refers to the strength of an external applied electric field to distort charge distribution (i.e., cause charge redistribution). For example, when light is used as an external field, the molecules undergo polarization and the velocity of the light decreases [58].

**Figure 5:**
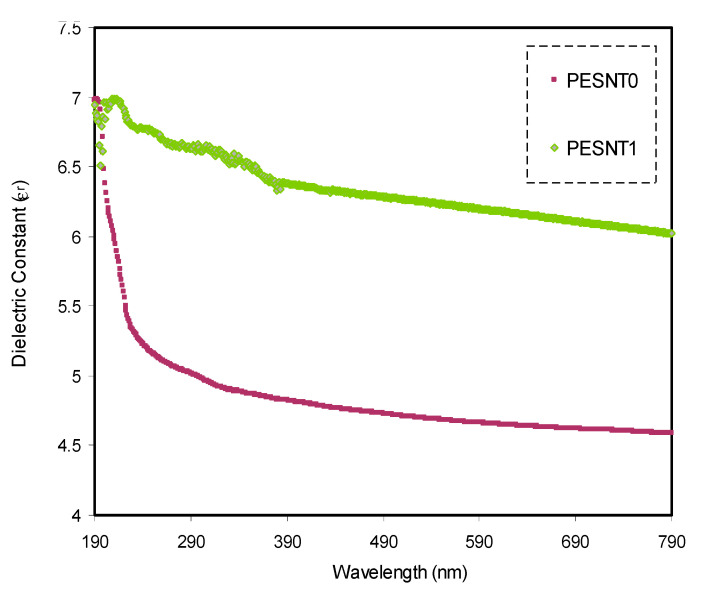
Optical dielectric loss (ε_r_) for pure PEO (PESNT0) and PEO:SnTiO_3_ (PESNT1) samples.

The parameter of the dielectric constant (ε_r_) is another important parameter in the characterization of the material’s optical properties. Figure 5 allows comparison of the optical parameters of ε_r_ of PEO/SnTiO_3_ and pure PEO. It was established that the parameter of ε_r_ is linked not just with the values of *n*, but also with values of *k*, as shown below [59]:

(3) $$\varepsilon_{r}=n^{2}-k^{2}$$

Accordingly, extrapolation of the optical ε_r_ plateau region to the axis of Y is employed to extract the optical ε_r_ of the PEO and 4 wt.% of PEO/SnTiO_3_ NC samples. It is apparent that the insertion of the SnTiO_3_ NPs produces an increment in the ε_r_ value from 4.5 to 6.3. The density of state’s increment is the cause of such an increment, as a straight connection exists between the density of states and ε_r_ through the polymer film’s forbidden gaps [46,48].

### 3.4. Band Gap Study

In the current work, the optical characteristics in terms of the complex optical dielectric function were plotted in order to gain a comprehensive understanding of the structure-property of samples. It is known that the complex dielectric function establishes a relationship between the applied electromagnetic field and the density of electrons [48]. Based on the quantum mechanics viewpoint, there is a substantial association between the material’s band structure and optical dielectric function. The real electronic transitions between the filled $\Psi_{K}^{V}$ and unfilled $\Psi_{K}^{C}$ wave functions cause photon absorption or emission and are represented by the optical dielectric loss (*ε*_i_). The expression below shows the optical parameter of *ε*_i_ [48,60,61]:

(4) $$\varepsilon_{i}=\frac{2\pi \;e^{2}}{\Omega \varepsilon_{0}}{\sum_{K,V,C}\left|\langle \Psi_{K}^{C}\left|\vec{u}•\vec{r}\right|\Psi_{K}^{V}\rangle \right|}^{2}\delta (E_{K}^{C}-E_{K}^{V}-\hbar \omega )$$

In Equation (4), ω stands for the frequency of incident photons and Ω stands for the volume of the crystal. The charge of an electron and permittivity in space are respectively referred to as e and $\varepsilon_{0}$. The position vector and a vector signifying the electromagnetic wave incident polarization are respectively referred to as $\vec{r}$ and $\vec{u}$. The CB and VB wave functions at *k* are respectively referred to as $\Psi_{K}^{V}$ and $\Psi_{K}^{C}$.

From a quantum perspective, the ε_i_ parameter is linked with the filled and unfilled materials’ electronic states, i.e., the optical energy band gap [62]. From the gained *n* and *k* data, the ε_i_ can be computed through the following relation [63]:

(5) $$\varepsilon_{i}=2nk$$

**Figure 6:**
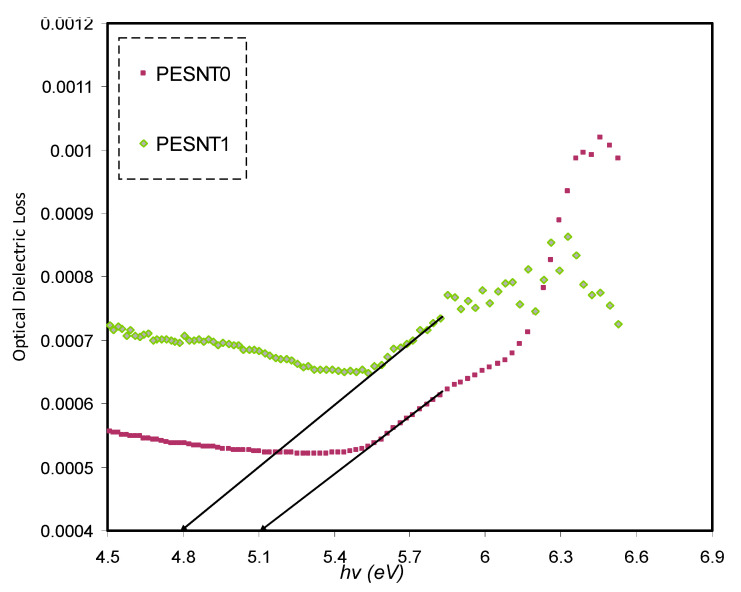
Optical ε_i_spectra of pure PEO (PESNT 0) and PESNT 1 films.

The ε_i_ as a function of *h*(υ) for pure PEO as well as PEO/SnTiO_3_ NC samples is shown in Figure 6. It was indicated that the optical band gap is computed on the basis of the extrapolated linear part intersection of ε_i_ in opposition to the *h*(υ) [63]. The electronic transition nature is precisely computed on the basis of Tauc’s method as well as ε_i_ plot.

**Figure 7:**
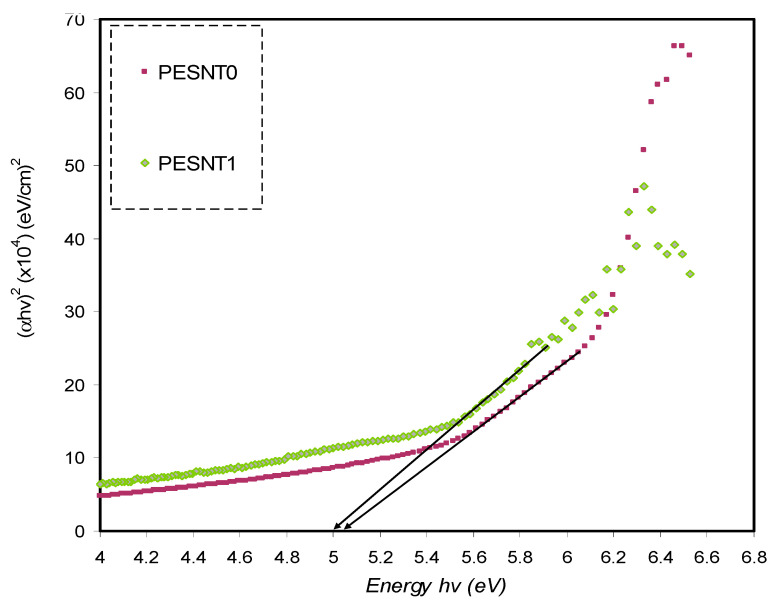
*(αhυ)^2^* in opposition to energy of photon for pure PEO (PESNT0) and doped (PESNT1) samples.

**Figure 8:**
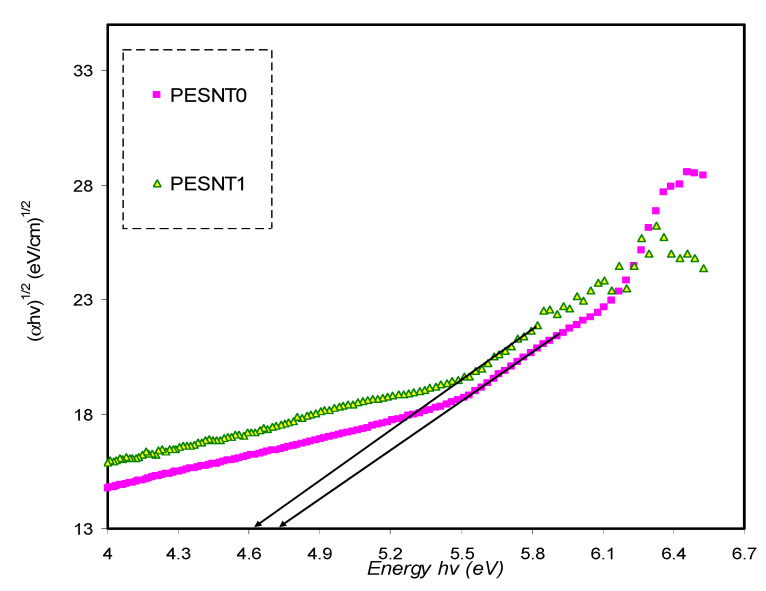
*(αhυ)^1/2^* in opposition to energy of photon for pure PEO (PESNT0) and doped (PESNT1) samples.

**Figure 9:**
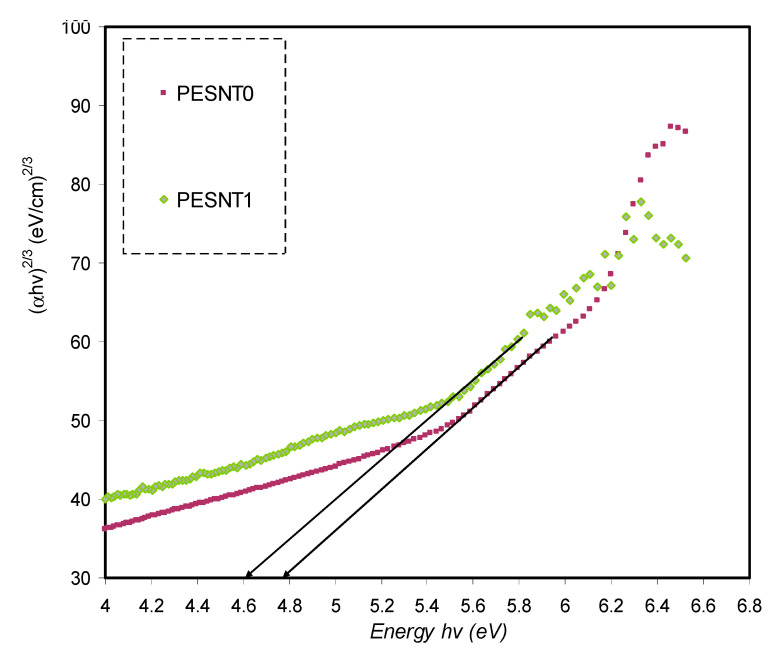
*(αhυ)^2/3^* in opposition to energy of photon for pure PEO (PESNT0) and PESNT1 composite samples.

**Figure 10:**
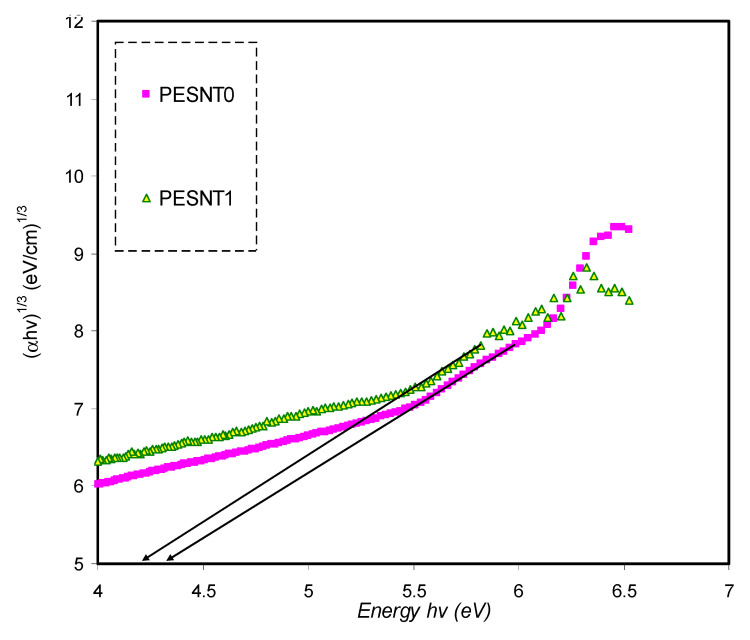
Plot of *(αhυ)^1/3^* in opposition to energy of photon for pure PEO (PESNT0) and PESNT1 composite samples.

Based on the analysis of the *α*, it is possible to calculate the optical energy band gap (E_g_) of the pure PEO as well as PEO/SnTiO_3_NC.To assess the energy gap of the films, Tauc’s relationship can be applied as shown below [6,50,64]:

(6) $$(\alpha h\upsilon )=B{\left(h\upsilon -E_{g}\right)}^{\gamma }$$

where *B* isthe constant inside the range of visible frequency and the *γ* exponent is employed in specifying the electron transitions nature and is reliant on the density of states distribution. *γ* = 2 or 1/2 to show indirect allowed transition and direct allowed transition, respectively. The situation is different for direct and indirect forbidden transitions, where γ is equal to 3/2 or 3, corresponding to direct forbidden transition and indirect forbidden transition, respectively [65,66]. Additionally, the (*αhν*)^1/γ^ plots in opposition to *hν* aid in ascertaining achievable transitions through extrapolating the graph’s straight line part on the axis of *hν* to *α* = 0 and consequently obtain the optical band gap. The (*hυα*)^1/γ^ plots in opposition to *hυ* for four values of *γ*, respectively, are shown in Figure 7, Figure 8, Figure 9 and Figure 10. The band gap values determined by Tauc’s method and plot of ε_i_ are tabulated in Table 1.

Table 1 clearly shows the optical band gap decrement resulting from the inclusion ofSnTiO_3_ NPs. It is suggested from the optical band gap decrement that the electronic structure of the PEO molecules undergo adjustments when SnTiO_3_ NPs are inserted, and defects produced in the PEO polymer might be the determinants of such adjustments [67]. Localized energy levels at the optical band gap might be created by those defects within the new bandgap states creation, mediating the electrons transition from the VB to the CB that underpins this decrement [6]. By comparing the estimated band gap energy and the energy estimated by the parameter of ε_i_, the nature of electronic transitions in the films can be identified [18]. By comparing the E_g_ acquired from Tauc’s method (Figure 7, Figure 8, Figure 9 and Figure 10) with the energy obtained from the ε_i_ plot (Figure 6), it becomes obvious that direct allowed (γ = 1/2) and forbidden (γ = 3/2) transitions are the main possible transitions for pure PEO and PEO/SnTiO_3_ NC, respectively. In applications such as light emitting photovoltaic diodes, as well as laser diodes, the best materials are most likely DBG materials. Previous studies established that PEO based polymer composites are significant for diverse optoelectronics applications including sensors, solar cells, transistors, diodes, capacitors, and energy storage [68,69].

## 4. Conclusions

The key conclusion of the current study is that the DBG polymer NC with amorphous structure is a vital material for applications in optoelectronic devices. The solution cast technique was employed for constructing PEO/SnTiO_3_ NC films.XRD examination exhibited that a clear interaction happened between SnTiO_3_ NPs and the PEO polymer. The addition of 4 wt.% SnTiO_3_ NPs emphasized the change of the PEO phase towards the amorphous phase. The optical band gap energy of pure PEO was modified by adding SnTiO_3_ NPs, as was calculated from UV-Vis spectroscopy analysis. This modification in the pure PEO’s band gap energy was evident from the interaction between PEO and SnTiO_3_ NPs. The bandgap reduction with increased amorphous region can significantly enhance the charge transportation within the polymer composite and make it a potential candidate for optoelectronic device applications including solar cells, optical waveguides, and LEDs. The type of electronic transitions between VB and CB was specified using the plot of *ε*_i_ and Tauc’s model. The *n* of PEO increased with addition of the SnTiO_3_NPs, as characterized by a plateau region at the high wavelength and a dispersion region at the low wavelength of incident photons. The optical parameter of ε_r_ also increased with addition of 4 wt.% SnTiO_3_NPs. This increment in ε_r_ was explained on the basis of formation of new energy levels in the middle of the VB and the CB as well as lowering of the band gap energy. The nature of electronic transitions in the pure and the composite material were identified from Tauc’s model. The optical *ε*_i_ examination was also carried out to calculate the optical band gap and establish the type of electronic transition.

## Figures and Tables

**Table 1:** Optical bandgap energy using Tauc’smodel as well as optical dielectric loss (ε_i_) plot.

Sample Code	E_g_ for γ = 1/2	E_g_ for γ = 2	E_g_ for γ = 3/2	E_g_ for γ = 3	E_g_ for ɛ_i_ Plot
PESNT 0	5.1	4.74	4.78	4.33	5.12
PESNT 1	5.00	4.61	4.612	4.21	4.78
